# 

**Published:** 1866-01

**Authors:** 


### Books and Pamphlets Received.

Circular No. 6, War Department, Surgeon Generals Office, Washington, Nov. 1, 1865. Reports on the Extent and Nature of the Materials available for the preparation of a Medical and Surgical History of the Rebellion. Printed for the Surgeon General's Office, by J. B. Lippincott & Co., Philadelphia.

Lectures on the Diseases of Infancy and Childhood; by Charles West, M. D., Fellow of the Royal College of Physicians; Physician to the Hospital for Sick Children. Fourth edition, from the fifth revised and enlarged English edition; Philadelphia: Henry C. Lea; 1866.

Obscure Diseases of the Brain and Mind; by Forbes Winslow, M. D., D. C. L., Oxon., &c., &c. Second American, from the third and revised English edition. Philadelphia: Henry C. Lea; 1866.

Rhinoscopy and Laryngoscopy; their value in Practical Medicine; by Dr. Friederich Semelder, Translated from the German, by Edward T. Caswell, M. D. New York: William Wood & Co., 61 Walker street; 1866.

Fifteenth Anniversary Meeting of the Illinois Medical Society, held in Bloomington, May 2d and 3d, 1865. Chicago: George H. Fergus; 1865.

Transactions of the Twentieth Annual Meeting of the Ohio State Medical Society, held at Ohio White Sulphur Springs, June 20th, 21st and 22d, 1865. Cincinnati: A Moore, 1865.

Council of Hygiene and Public Health; Citizen's Association of New York. Report on Epidemic Cholera. New York: Sanford, Harroun & Co., 1865.

